# Adapting the Donabedian model in undergraduate nursing education: a modified Delphi study

**DOI:** 10.1186/s12909-024-05187-7

**Published:** 2024-02-27

**Authors:** Marjan Ghofrani, Leila Valizadeh, Vahid Zamanzadeh, Akram Ghahramanian, Ali Janati, Fariba Taleghani

**Affiliations:** 1 Department of Pediatric Nursing, School of Nursing and Midwifery, Tabriz University of Medical Sciences, Tabriz, Iran; 2 Department of Pediatric Nursing, School of Nursing and Midwifery, Shahid Beheshti University of Medical Sciences, Tehran, Iran; 3 Department of Medical Surgical Nursing, School of Nursing and Midwifery, Shahid Beheshti University of Medical Sciences, Tehran, Iran; 4 Medical Education Research Center, Health Management and Safety Promotion Research Institute, Tabriz University of Medical Sciences, Tabriz, Iran; 5 Department of Medical Surgical Nursing, School of Nursing and Midwifery, Tabriz University of Medical Sciences, Tabriz, Iran; 6 Department of Health Service Management, School of Management and Medical Informatics, Tabriz University of Medical Sciences, Tabriz, Iran; 7 Nursing & Midwifery Care Research Center, School of Nursing & Midwifery, Isfahan University of Medical Sciences, Isfahan, Iran

**Keywords:** Education, Nursing, Delphi technique, Donabedian model, Undergraduate Program Evaluation

## Abstract

**Background:**

Donabedian conceptual and multidimensional framework has been used in several studies in an educational context. In this study, we aimed to adapt the Donabedian three-component model (structure, process, and outcome) in undergraduate nursing education. This conceptual framework provides a comprehensive image of nursing education institutions and can help to evaluate institutions by measuring different aspects of their performance. A comprehensive understanding of the various elements of an educational institution helps to develop a complete, appropriate relevant set of performance indicators.

**Methods:**

This was a modified Delphi study. It had three rounds. The expert panel consisted of nursing faculty members and nursing Ph.D. students. In the first round, a questionnaire was designed based on interviews, focus groups, and a literature review. Experts rated their agreement with each element on a 5-point Likert scale in rounds two and three. The consensus level was set as 75%. The stability between rounds was also determined by calculating kappa coefficients. One Sample T-Test was also calculated for new items in round three.

**Results:**

All 55 items of the questionnaire were confirmed in the second round based on the consensus percentage of 75. Five new items were added to the third round based on comments in round two. Eventually, all elements except one were confirmed according to the consensus level, kappa values, means, and One-Sample T-Test in round three. The structure's key elements include staff (academic and non-academic); equipment; guidelines; resources and facilities; and students’ demographics and characteristics. Process key elements include communication; education; evaluation; cooperation; and consultation. Outcome key elements include knowledge development; nursing image; alumni’s outcome; students’ outcome; related medical centers’ performance; accreditation and evaluation results; and satisfaction.

**Conclusions:**

Different elements of a nursing education institution at the bachelor's level were determined. The results of this study can help related bodies to develop and implement a comprehensive and systematic evaluation. These results can also be a basis for making this model useful in other nursing courses or education in other fields.

**Supplementary Information:**

The online version contains supplementary material available at 10.1186/s12909-024-05187-7.

## Background

Nurses contribute to global health [1] and affect patient’s experience of care [2]. The continuum of nursing practice contains a broad range from health promotion, to disease prevention, to coordination of care, to cure, and to palliative care [3]. They even deal with the private and personal aspects of people's lives [4]. Nursing care covers all aspects of health needs containing physical social, mental, and spiritual [3].

To improve health care and health systems, nursing education must be improved [5]. Improved education systems ensure quality care provided by graduated nurses [3]. The production of competent nurses is the critical role of nursing education [5]. For improving the quality of higher education institutions, evidence-based evaluation and audit are needed [6]. Audits, evaluations, and assessments have become increasingly common in higher education [7]. These can be complex, interrelated processes in nursing education [8]. One of the practical challenges in conducting evaluation is the use of appropriate evaluation methods and tools [9].

Most educational institutions use peer review and accreditation as an assessment of their performance [10]. Accreditation is often cited as a barrier to program innovation [11], it can be complex and time-consuming [12], and understanding it’s outcomes is difficult for lay stakeholders [13]. Performance measurement can be used as a powerful tool for evaluating and controlling organizations [14]. Even if accreditation is used, it is useful to have performance indicators to evaluate the accreditation program [15, 16].

Performance measurement includes efforts to monitor, evaluate, and establish the relationship between the organization’s goals, resources, and activities, with the results, outputs, and achievements of goals [17]. Appropriate indicators must be selected to measure the performances [18]. Key performance indicators provide a powerful and effective tool to evaluate, adjust, and motivate employees to work more intensively, thus bringing more benefits to the common cause [19].

During the previous steps of this project, we were looking to develop performance indicators for nursing education institutions. We held several interviews and focus groups with different groups of stakeholders. We also conducted a review of existing indicators on nursing schools’ websites. During the data analysis, we realized that most of the indicators or areas that need to be measured can be classified based on three components: structure, process, and outcome. We were also looking for a proper conceptual framework that is compatible with the study. It is useful to have a conceptual framework to guide the development of a set of indicators [20]. We needed a conceptual framework to help us in developing and organizing the indicators. Existing frameworks were reviewed to find a suitable framework for the study. We also used a published systematic review of existing frameworks [21]. During this review and our data analysis, we found that the Donabedian Model is completely compatible with the collected data and could be used as the conceptual framework in indicator development for nursing education institutions. So we used the Donabedian quality improvement model as a guide to classify data. However, this model was used in educational contexts before, but it has never been adapted comprehensively in an educational context. We assumed maybe adapting this model and providing its elements in nursing education may be a basis for further studies or help in quality improvement studies.

Avedis Donabedian proposed a framework in the 1960s. Based on his framework the quality of health care could be assessed by evaluating three elements—structure, process, and outcome. Its flexibility makes it useful in quality improvement initiatives across clinical settings [22]. The Donabedian model provides a framework for conducting evidence-based service evaluation. With this model, the differences between elements of structure, process, and the outcome can be recognized. This would provide a more complete picture of a service [23]. By following Donabedian's model, the discovery of a beneficial outcome will be of much practical value because one knows what factors are necessary for it to happen (structure) and how it was actually achieved (process). In this manner the evaluation of quality can be more concrete and comprehensive [24]. According to Donabedian’s model, improvements in the structure can improve processes that should in turn improve outcome [25].

Donabedian conceptual and multidimensional framework has been frequently used [26]. Including several studies in the educational context [24, 27–30]. But we could not find a study that has adopted this model in nursing education. In this Delphi study, we aimed to adapt the Donabedian model as a proper conceptual framework for nursing education. We specified its elements comprehensively and defined each of its three components. Later this conceptual model could be used for performance indicator development in undergraduate nursing education or other goals.

## Methods

### Design

**Fig. 1:**
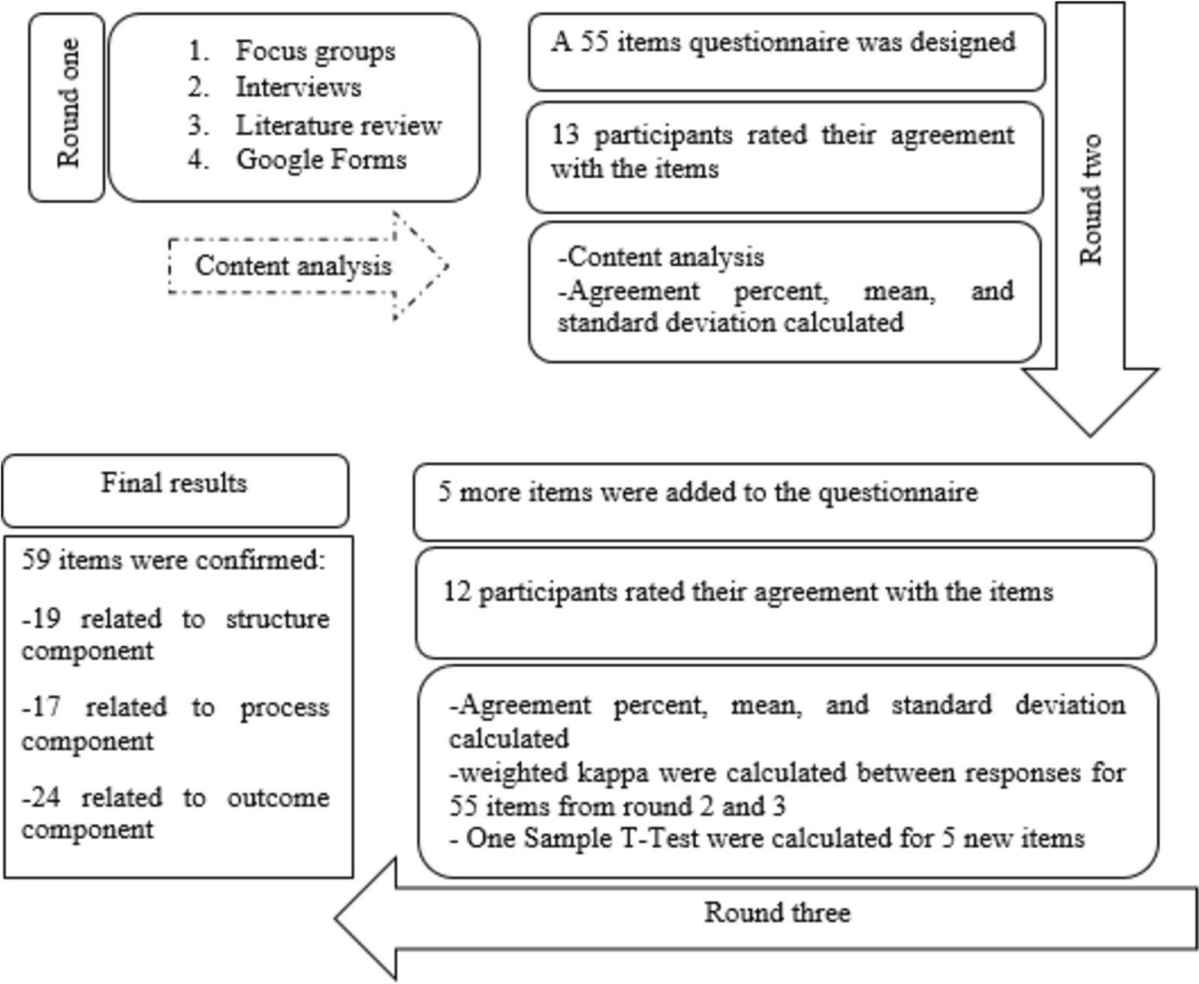
Schematic view of the Delphi rounds. This modified Delphi study had three rounds, in round 1 a questionnaire was designed that were rated by expert panel members in round 2 and 3. 5 New items were added to the questionnaire based on expert panel opinion in round three. Finally, 59 items were confirmed

This is a Modified Delphi study. The Delphi method assumes that group opinion is more valid than individual opinion and the technique aims to achieve agreement among a group of experts on a certain issue [31]. Expert consensus methods are commonly employed in consumer, education, and health services research [32]. This Delphi study had three rounds. Delphi studies are typically carried out in two to three rounds [33]. It is difficult to keep a high response rate within a Delphi study that has many rounds [31]. A schematic view of the Delphi rounds is provided in Fig. 1.

### Preparation phase

We considered the main components of the Donabedian model (structure, process, outcome) and tried to identify and confirm the elements of these three components during the Delphi rounds. Like the Donabedian, we assumed that the structure would affect the process and the process would affect the outcome [34]. Also, based on Donabedian's definitions of structure, process, and outcome [35], we considered the following definitions for these three components in nursing education (these definitions were also confirmed by the experts during the Delphi rounds):

- Structure: Attributes which in and with the nursing education can occur. It includes all factors affecting the conditions of education.
- Process: All the acts of nursing education institutions.
- Outcome: All the effects of nursing education institutions.

### Expert panel

The expert panel for this study consisted of the nursing faculty members and the nursing Ph.D. students. Regarding the faculty members, we tried to invite experts with experiences in different areas. They consisted of those who had experiences in nursing school management, were members of the board of nursing, or simply were nursing professors or instructors. Regarding the Ph.D. students, besides they were students (as an important group of stakeholders) and familiar with the concept of model adaptation (as they were at the Ph.D. level), we invited those who had experiences in nursing education or practice. The expert is defined as a person with knowledge and experience in a particular subject matter. Experts are used to increase the qualitative strength of recommendations or consensus. There is no standard size of the panel members, but 10–15 experts are considered acceptable [36, 37].

### Sampling

Purposive sampling was used in this study. People who were considered "experts" in the field of the study, and also had a variety of experiences, were selected and invited to participate in the study. Purposive sampling is designed to find people who can and are willing to provide information through knowledge or experience [38].

### Setting

This study was based in a nursing and midwifery faculty. But experts from colleges across the country were invited to study as the expert panel members.

### Main features of Delphi studies

**Fig. 2:**
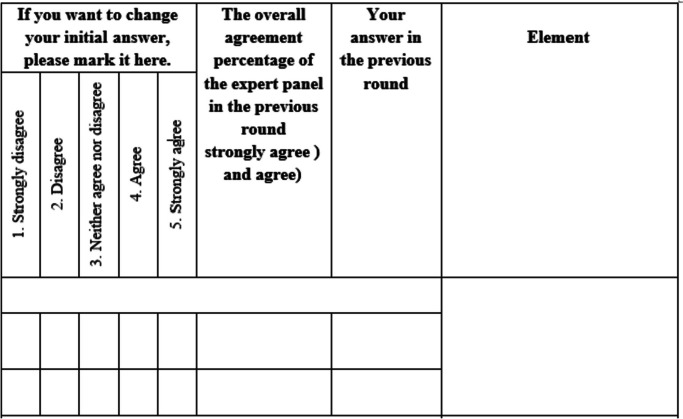
The questionnaire that were designed for the third round of Delphi. This questionnaire provides expert previous rate on the elopements, expert panel overall agreement, and a chance for experts to change their previous answers

The Delphi method presents characteristics and features including the anonymity of experts, iteration, controlled feedback, and qualitative and quantitative data [39]. In this study, these features were addressed in different rounds:

- Anonymity of experts: In this study, expert panel members were anonymous to each other. This assures the free expression of opinions and helps to avoid social pressure from dominant individuals [40].
- Iteration: It is possible for experts to change their opinions or judgments at any point in the study [40]. We prepared an exclusive questionnaire for each of the participants in the third round (Fig. 2). In these questionnaires, there was a section that specified the person's answer to each of the items in the previous round, and an opportunity was created for the expert to change her/his previous answer if she/he wished.
- Controlled feedback: Experts are informed about the views of other experts who participate in the study [40]. In the third round’s questionnaire, besides each person’s own answer to each of the items in round two, we also provided the overall expert panel members’ agreement percentage for each specific item. So the panel members could know what had been the overall expert panel opinion about the item.
- Although the Delphi method is qualitative, it can provide quantitative results [40]. In this study, the qualitative data that was retrieved from the first round and also from comments in the second round were converted to items and were rated quantitatively by the expert panel.

### Consensus

The panel of experts was asked to indicate their level of agreement with each of the specified elements in a 5-point Likert scale (5. Strongly agree, 4. Agree, 3. Neither agree nor disagree, 2. Disagree, 1. Strongly disagree). Two "Strongly agree" and "Agree" answers were considered as agreement, the answer "neither agree nor disagree" as neutral and the answers "Disagree" and "Strongly disagree" were considered as disagreement. The level of agreement for achieving consensus was set as 75% [41]. This means that at least 75% of experts had to rate an element 5 or 4, till that item can be maintained. Besides this we also calculated the stability of responses between rounds 2 and 3 to decide on ending the Delphi rounds [41, 42].

### Round one

In the modified Delphi technique, the first round can be made up of focus groups or face-to-face interviews or a structured questionnaire may be used, with quantitative questions based on the literature in previous researches [43].

In this round, a questionnaire was designed consisting of 55 items of expected elements for the three components of structure, process, and outcome. These elements were the result of the literature review, the interviews, and the focus groups that were held with stakeholders in previous steps of the main study that this Delphi study is part of. Our aim in the main study was to develop nursing education key performance indicators. We wanted to know about the aspects or indicators that should be measured to represent a nursing education institution’s performance. We tried to gather different groups of stakeholders’ perspectives. Four focus groups were conducted with bachelor's, master's, and Ph.D. students (total number of students = 27). A focus group was conducted with nurses (*n* = 6). Seven individual interviews were conducted with nursing faculty members and policymakers. Also, an online form was designed on Google Form, which was filled by 91 nurses, nursing students, and nursing faculty members (demographic characteristics of the participants and further information about this part are provided in the supplementary file named “Additional information about the major study”).

In the first round of this modified Delphi study, a questionnaire was designed from the findings of these steps. This questionnaire was later rated in the subsequent rounds by the expert panel’s members. Except for the questionnaire items (elements) we also prepared a section for each of the three components so that the expert panel members could provide further comments about items or possible other elements that we hadn’t mentioned. We also prepared an extra section where the expert panel could leave their comments about the entire structure of the model designed based on the Donabedian Model. At the beginning of the questionnaire we provided some explanation about the study aims, the Donabedian Model, how the items (elements of three components) were developed, and how the questionnaire must be filled. We explained the components of the Donabedian model and their definitions in the main model. Then we provided explanations about the previous steps of the study, including interviews and literature review, and how the elements related to each component were obtained. We also stated some reasons regarding the use of this model in educational fields and the importance of adapting the model in nursing education. Then the participants were asked to express their level of agreement with the fact that each of these mentioned elements can be part of the structure, process or outcome of a nursing education institution. In other words, we wanted to ensure with the help of experts that the elements considered for each component are correct and relevant.

### Round two

After designing the questionnaire in the first round, in the second round of this Delphi study, 20 experts were invited to participate in the study. Of these, 15 experts agreed to participate. If possible, the questionnaires were delivered to the experts in person, and if they were not available, questionnaire were delivered to them by email. If necessary, reminder messages were sent to some of the participants one to three times, and finally, 13 members answered the questionnaire in the second round of the study. We put one-month limitation to complete this round.

### Round three

In the third round, after editing the questionnaire of the second round based on the panel members’ comments, the edited questionnaire was given to thirteen panel members and they were asked to rate the items. Their previous answer and feedback from other expert panel members’ answers were provided. By sending reminder messages, finally, after the completion of one month considered for the third round, 12 questionnaires were collected. At the beginning of the third round’s questionnaire, we provided some explanation about the study again, also how the agreement percent were calculated, which items (elements) were new and which had been edited.

### Data analysis

Response rates were calculated for rounds (Table 1). Besides the fact that no specific guidelines exist for acceptable response rates for Delphi studies and that response rate varies from 8 to 100% in studies. Several authors recommend that a 70% response rate for each round is necessary to maintain accuracy [31].

**Table 1:** Response rates for two rounds of Delphi. The ratio of the filled questionnaire to the people invited to the study was calculated in each round

Round	Invited members	Those who accepted to participate	Those who filled the questionnaire on time	Response rate
Two	20	15	13	65%
Three	13	13	12	92.3%

Qualitative data from round two were analyzed by content analysis. About ratings that were assigned to each element, quantitative data analysis was conducted. SPSS version 23 was used. Mean, Standard deviation, and agreement percent (5. Strongly agree plus 4. Agree) for each of the elements calculated (Table 2).

**Table 2:** Agreement percent, standard deviation, mean, kappa value, and one sample T-Test *p*- values for elements of structure, process, and outcome in undergraduate nursing education

Elements	Second round			Third round			The stability between rounds 2 and (Weighted Kappa)	One Sample T-Test (*P*-value)
	Agreement percent (%)^a^	Std. Deviation	Mean	Agreement percent (%)^a^	Std Deviation	Mean		
1^b^	91.7	16-Jan	4.5	91.7	0/66	4.42	0.71	-
2	100	0/45	4.75	100	0/49	4.67	0.80	-
3^c^	-		-	81.9	0/75	4.18	-	0.00
4	100	0/38	4.83	100	0/38	4.83	0/4	-
5	100	0/38	4.83	100	0/00	5	0	-
6	91.6	0/62	4.75	91.6	0/62	4.75	1	-
7	100	0/00	May-00	100	0/28	4.92	0	
8	100	0/38	4.83	100	0/28	4.92	0/625	-
9	100	0/38	4.92	100	0/28	4.83	0/625	-
10	100	0/38	4.83	100	0/38	4.83	1	-
11	91.7	0/90	4.58	100	0/49	4.67	0/72	-
12	100	0/38	4.83	100	0/38	4.83	1	-
13	100	0/28	4.92	100	0/28	4.92	1	-
14	100	0/28	4.92	100	0/28	4.92	1	-
15	100	0/45	4.75	100	0/45	4.75	1	-
16	100	0/38	4.83	100	0/38	4.83	1	-
17	-	-	-	100	0	5	-	-
18	-	-	-	83.3	0.79	4.42	-	0
19	87.5	0.74	4.38	91.6	0.9	4.42	0.39	-
20	100	0.51	4.58	100	0.51	4.58	1	-
21	91.7	0.66	4.58	91.7	0.66	4.58	1	-
22	-	-	-	83.3	1	4.5	-	0
23	100	0.51	4.58	100	0.51	4.58	1	-
24	100	0.38	4.83	100	0.38	4.83	1	-
25	100	0.38	4.83	100	0.28	4.92	0.62	-
26	100	0	5	100	0	5	0	-
27	91.6	0.62	4.75	91.7	0.65	4.67	0/88	-
28	100	0.38	4.83	100	0.28	4.92	0.62	-
29	100	0.38	4.83	100	0.28	4.92	0.62	-
30	100	0.38	4.83	100	0.28	4.92	0.62	-
31	100	0.49	4.67	100	0.45	4.75	0.80	-
32	100	0.46	4.73	100	0.49	4.67	1	-
33	100	0.51	4.58	100	0.51	4.58	1	-
34	100	0.49	4.67	100	0.51	4.58	0.82	-
35	100	0.28	4.92	100	0.28	4.92	1	-
36	91.6	0.62	4.75	91.6	0.62	4.75	1	-
37	91.7	0.65	4.67	83.4	0.99	4.42	0.44	-
38	100	0.45	4.75	100	0.49	4.67	0/80	-
39	91.7	0.65	4.67	91.7	0.65	4.67	1	-
40	100	0.51	4.63	75	0.78	4.27	1	-
41	100	0	5	100	0	5	0	-
42	100	0	5	100	0	5	0	-
43	91.7	0.65	4.67	91.7	0.65	4.67	1	-
44	75	0.9	4.42	75	0.9	4.42	1	-
45	100	0.45	4.75	100	0.49	4.67	0.80	-
46	91.6	0.67	4.5	91.7	0.66	4.58	0.90	-
47	100	0.3	4.91	100	0.28	4.92	1	-
48	91.6	0.67	4.5	91.6	0.67	4.5	1	-
49	100	0.38	4.83	100	0.38	4.83	1	-
50	100	0.49	4.67	100	0.49	4.67	1	-
51	100	0.45	4.75	100	0.45	4.75	1	-
52	-	-	-	72.8	0.94	3.91	-	0
53	75	1.08	4.42	83.3	1	4.45	0.95	-
54	100	0	5	91.7	0.57	4.83	0	-
55	100	0.45	4.75	100	0.45	4.75	1	-
56	83.3	0.98	4.33	75	1.03	4.17	0.91	-
57	100	0.28	4.92	100	0.28	4.92	1	-
58	100	0.28	4.92	100	0.28	4.92	1	-
59	100	0.28	4.92	100	0.28	4.92	1	-
60	90.9	0.90	4.73	83.4	1.15	4.33	0.76	-

^a^The percentage of agreement is the sum percent of two scores of 4 and 5 on the 5-point Likert scale

^b^Items number 1, 4, 14, and 19 were edited based on expert panel members’ comments in round two

^c^Items number 3, 17, 18, 22, and 52 were added to round 3 and were created based on panel members’ comments in round 2, so instead of the kappa coefficient, One Sample T-Test was calculated for them

Weighted Kappa coefficient were also calculated by R software to assess responses stability between rounds 2 and 3. For new item that were added to the round three based on panel members comment, One Sample T-Test were calculated (Table 2).

## Results

### Demographic characteristics

We had 13 members in round 2 and 12 members in round 3. There were seven faculty members and six Ph.D. student. Six of the faculty members had Ph.D. degree too. The average year of experiences were 19.3. The participants’ experiences included: Scientific nursing associations membership; Evaluation and accreditation committee’s membership; Nursing Board membership; membership of the University Nursing Policy Council; membership of the National Nursing Research Network; Education development office (EDO) director; Nursing research centers membership; Hospital and school management; Nursing; and Nursing education.

### Rounds statistics

#### Round 2

According to the consensus level of 75%, the consensus was reached for all the items in round 2.

#### Round 3

The consensus level of 75% was reached for all the items in round 3 except item number 52 (Student transfer). This item had been added to round 3 based on the comments in round 2.

#### Weighted kappa

Cohen suggested the Kappa result be interpreted as follows: values ≤ 0 as indicating no agreement and 0.01–0.20 as none to slight, 0.21–0.40 as fair, 0.41– 0.60 as moderate, 0.61–0.80 as substantial, and 0.81–1.00 as almost perfect agreement [44]. Forty-six items got kappa greater than 0.6 which is a substantial or perfect agreement.

For items number 5, 7, 26, 41, 42, and 54 the kappa coefficient got 0 value that is because at least one of the variables is constant and has zero variance, because of this the kappa could not be calculated. This doesn’t mean that there is no agreement between two rounds answers for these items. These items got a high percentage of agreement and high means, for this reason, we didn’t omit these items.

For items, number 4, 19, and 37, kappa values are 0.4, 0.39, and 0.44 respectively. Sometimes high agreement and low kappa values are the results of the first kappa paradox [45]. Because they had an acceptable level of agreement percent and means, and kappa was also fair or moderate we kept these items too.

#### One sample T-test

One Sample T-Test was calculated for 5 new items in the third round. Except for item number 17, which T-Test could not be calculated due to zero variance, the p-value was statistically significant for the remaining 4 items (Table 2). For item number 17, because the agreement percent is 100, and the mean is 5, this element was also maintained. For the other 4 items, the mean is also more than three (the maximum value could be 5(.

#### Final result

**Fig. 3:**
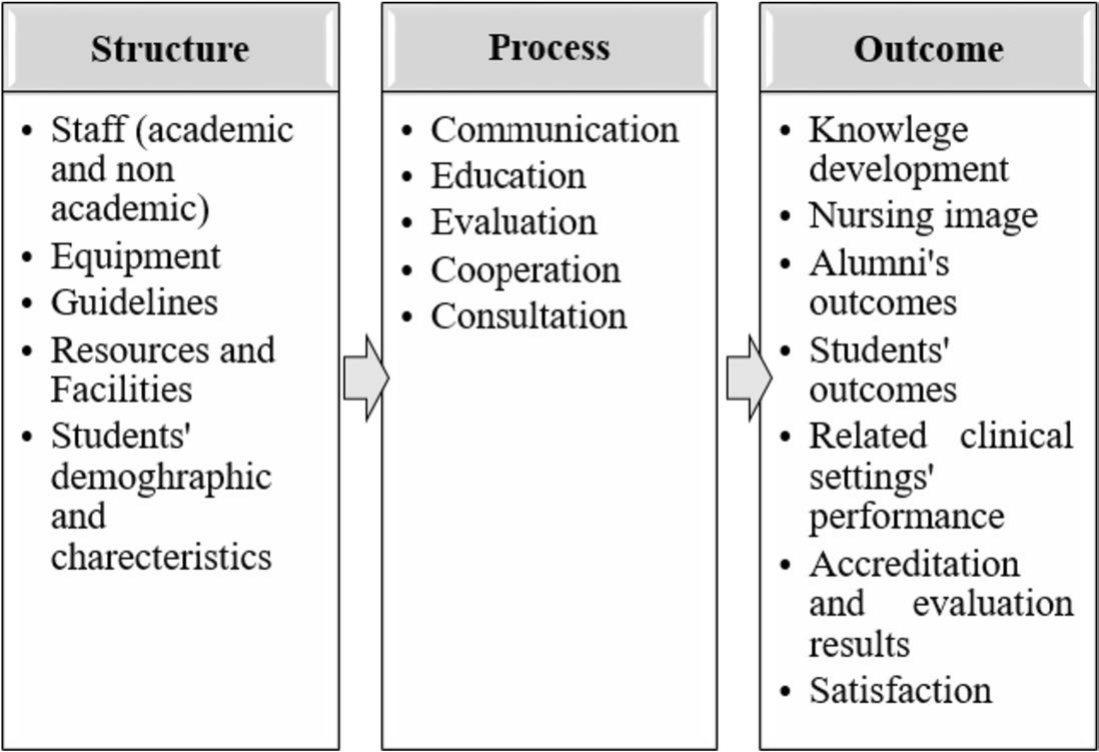
Key elements of structure, process and outcome in undergraduate nursing education. These elements were confirmed during Delphi round for undergraduate nursing education

According to the percentage of agreement, kappa values, means, and T-test, all the elements except element number 52 (students transfer) are confirmed. Structure’s, process’, and outcome’s key related elements are presented in Fig. 3 (items related to each of these key elements are available via a Supplementary file named “Model”).

In this study, elements of a nursing education institution performance from the perspective of different stakeholders were confirmed by nursing experts and nursing education experts.

##### Structure

Five main elements were determined for the structure component. According to the definition of the structure component in this model, all the features that nursing education occurs in or with them can be part of the structure of this model. The employees, whether they are involved in education or those who are responsible for other tasks related to an educational institution, can be one of the key elements of structure and undeniably contribute to the institution performance.

Maintaining the human resources standards, along with the training workshops that are held for them, can help improve their performance and ultimately improve the performance of educational institutions. In exchange for the proper performance, reward and payment systems can be a motivation for employees or even a basis for attracting more skillful and competence employees to the institution, and in turn improve the institution performance.

Equipment, guidelines, resources and facilities, along with staff, can help educational institutions achieve their desired goals. Available facilities and equipment, educational spaces, and educational aids can effect on students and staff performance and as a result the total performance of institution.

Although the outcomes related to the students and graduates of an institution can be the functional consequences of an institution, the basic characteristics of the students who enroll in an educational institution can be its structural components. Because these initial specifications can affect the process and ultimately the achieved outcomes. On the other hand, the performance of the institution itself will be effective in attracting students with special characteristics to the institution.

##### Process

The processes include all the acts of an educational institution, if they are implemented well, they can lead to the desired outcomes by using the existing good structure. To establish desirable performance or improve it, an organization needs different acts either inside the organization or beyond. Each of these acts can help achieve desired outcomes by attracting resources, producing knowledge, presenting knowledge, or introducing the organization to outside world. In this study, the processes included communication, education, evaluation, collaboration, and consultation.

An educational institution with good performance will usually be able to take a step by establishing communication either at the level of the organization or beyond the organization; national and international cooperation in line with its strategic goals, or even the promotion of the nursing field. The organization must establish proper communication and collaboration with employees, stakeholders, other institutions, or related bodies. The educational organization should transfer their valuable experiences for use in appropriate cooperation with the industry, other schools, or other related centers outside the faculty and also use these organizations information and experiences. The school and these organizations should work together to produce products, services, or knowledge in order to achieve their lofty organizational goals and also benefit the country, and even the national and international community.

Sufficient quality education should be provided to students in connection with all their educational and information needs. When necessary, student should have access to appropriate individual consultation, and finally the organization, staff, and students should be evaluated appropriately.

##### Outcome

Participants expected outcomes included factors related to stakeholders and diverse audiences. They believed that a nursing education institution should contribute to the production of knowledge related to the field of nursing and also affect the image of nursing in a society. From the perspective of the participants in this study, the performance of nursing education institutions will have consequences for their students and graduates, which include a range of issues such as knowledge, skills, higher education, professional competence, and even their interest in the field in which they are studying.

Also, medical centers related to these educational centers can be affected by the performance of the school. Alumni with appropriate skills, training, and education can help improve the performance of healthcare institutions. School and faculty members’ collaborations with these settings can be helpful in different ways.

The results of external evaluations and accreditation conducted on the school can be the result of the performance of the nursing education institution, also the satisfaction of staff, service recipients, and employers is important as they benefit from the performance of the institution.

## Discussion

This is a Modified Delphi study that is part of a greater study that aimed to develop undergraduate nursing education key performance indicators. In this part of the study, we aimed to adapt the Donabedian Model for use in nursing education. We tried to specify its elements comprehensively and define each of its three components.

We needed a conceptual framework to get an overview of nursing education institutions in order to measure their performance. By examining the existing frameworks as well as the findings from the previous steps of the study, we realized that we need a framework that can organize our data into three components: structure, process, and outcome. This framework exists in healthcare with the aim of measuring the quality of their performance (Donabedian model). We decided to adapt the Donabedian Model. Its content can be useful to design performance measurement systems or improve performance in education. Many of the frameworks we reviewed were designed for healthcare, and some of the frameworks designed in education were not appropriate or too complex to organize our findings. Donabedian's framework has defined the main components of an organization with a simple yet almost complete design. The relationships between the components are clear. The emphasis of this model is on performance measurement, which was the main goal of our main project and It was consistent with our data.

The main elements obtained for each of the components of the Donabedian model in nursing education included 5 elements for structure |(staff, equipment, guidelines, resources and facilities, students’ demographic and characteristics), 5 elements for process (communication, education, evaluation, cooperation, consultation), and 7 elements for outcome component (knowledge development, nursing image, alumni’s outcome, students’ outcomes, related clinical settings performance, accreditation and evaluation results, satisfaction.

In this study, we adapted a healthcare model for use in an educational context. In a study by Selvik et al. also a health care model were served well as a conceptual framework for analyzing the benefits of educational efforts [46]. In Custis et al.’s study, through the use of Donabedian’s framework, the healthcare informatics students completed essential learning activities [47]. In another study, the structure and process of surgical oncology patient education within one integrated health system were assessed using the Donabedian framework [48]. Avci in his dissertation utilized Donabedian’s three approaches to formulate quality standards and indicators in bioethics education [30]. The results of these studies confirm the fact that the models designed in certain contexts can be generalized and used in other contexts. Conceptual frameworks can be used for different purposes in education. Considering the purpose we have of using a conceptual framework, we can use the framework designed in other disciplines and there will be no need to design a new framework. We needed a conceptual framework that could clarify all aspects of a nursing education institution and show us a clear and comprehensive picture of this interesting phenomenon. so that we can use this image for evaluation of these institutions. For this reason, the use of Donabedian's framework, which is designed to evaluate the performance of institutions, seems appropriate. It was only necessary to adapt this framework in the new context.

A study by Anderson et al. discusses that Joint Commission on Accreditation of Healthcare Organizations (JCAHO) framework can be adapted to evaluate the quality of nursing programs and it could be applied in an academic setting to improve student and organizational outcomes [49]. In a study by Lippe and Carter, Stufflebeam's Context, Input, Process, and Product model (CIPP) was utilized to evaluate end of life care content in a nursing education program. They concluded that model serves as a valuable guide for curriculum evaluation [50]. As these studies concluded, the conceptual frameworks of other disciplines could be well used in nursing education.

We used the Delphi technique as the means of model adaptation. The Delphi method is widely used to adapt or develop models in different contexts. In Boyer’s study, a modified Delphi method supported the adaptation and validation of a nursing competencies framework [51]. In another study, the Delphi method was used to develop a process model of key elements in the implementation process [52]. In Stockton et al. study, to identify key elements of a framework for the adaptation of specialist community-based child and family health, a Delphi study was conducted [53]. There are numbers of studies that have used different kinds of the Delphi techniques to develop or adapt models and frameworks. It seems that this method can be proper for achieving the goals of such studies. Because the first round of this Delphi study is based on interviews, focus groups, and literature review we used the modified Delphi technique as the study method [43].

We specified elements of each of the Donabedian model’s three components in nursing education. The first component is structure. In our study, staff (academic and non-academic), equipment, guidelines, resources and facilities, and students’ demographics and characteristics are key elements of this component in nursing education. In a study by Cassiani et al., in the designed instrument based on the Donabedian model to assess the situation of nursing education, the items related to structure in the instrument assessed the numbers of students and faculty, school policies or guidelines, classrooms, and laboratories [54]. In another study, structure expectations for critical care nurse education included a standard core curriculum, clinically credible academic staff, and courses compliant with a higher education framework. Published workforce standards and policies were important structures for the practice learning environment [55]. In a study by Jarrar et al., the resources (materials, facilities, and humans) as well as the organizational structure, policies, and procedures reflect the structural quality [56]. The findings of these studies regarding structure is compatible with our study findings. The findings of these studies together with our study show that human resources, equipment, facilities, physical space and guides are among the elements that are considered in relation to an educational institution.

In our study, process key elements include communication; education; evaluation; cooperation; and consultation. In Cassiani et al.’s study, the items related to the process included general professional competencies, curriculum model and teaching/learning strategies, clinical experiences, nursing program evaluation, and student evaluation [54]. This shows that education and evaluation are among key elements in both studies.

Based on our findings, outcome key elements include knowledge development; nursing image; alumni’s outcome; students’ outcome; related medical centers’ performance; accreditation and evaluation results; and satisfaction. In Cassiani et al.’s study, outcomes or results focused on the place of employment of the graduates, and whether they were integrating competencies related to Universal Health in their work [54].

The findings of this study can provide a framework for policymakers or nursing managers to systematically evaluate their educational institutions. This adapted model provides a comprehensive set of nursing education institution elements and helps to design a performance measurement or evaluation system. These results can also be a basis for making this model useful in other nursing courses or other fields. We believe this study may be the first study that has comprehensively adapted the Donabedian Model for use in nursing education.

The faculty can achieve a more comprehensive view of the faculty and its performance by using the elements identified in this study in internal evaluations. During the evaluation, taking into account the important areas from the point of view of the main stakeholders of the college and identifying and fixing the weak points of these areas, it can gain a higher position in today's competitive market.

The findings of the study by presenting in national or international conferences and scientific assemblies can help policy makers in nursing and similar clinical disciplines so that they can use the ideas presented in this study and the improvement model for performance evaluation and performance improvement systems. The quality of Donabedian should be helped and by integrating and completing the findings of this study and other studies, they should take steps to improve the performance of their institution and the nursing profession.

This study was carried out with the help and participation of various groups of nursing education stakeholders, as well as the use of numerous texts and websites of various nursing schools around the world. However, it seems that consultation with other stakeholders and professionals in the international fields of nursing education, or newer fields related to higher education such as sustainable development, should be given more attention.

Also, this study was done with an emphasis on nursing education at the undergraduate level, and probably other elements in graduate level of education may need to be considered.

### Study limitations and strengths

- One of the limitations of our study was that the response rate in the second round was 65%. Although it is better to keep the response rate at least 70% in all rounds, but because the response rate was significant in the third round, this rate can be attributed to the reluctance of people to participate in the study or ignoring the invitation to participate from experts who were invited via email, Because the response rate in the first round for the experts who were invited in person was 100%.
- Another limitation is that because 5 items were new in round 3 we couldn’t calculate kappa for them. Continuing the Delphi rounds for these 5 items might decrease the response rate, and it also demanded more time.
- One of the strengths of this study is the using different groups of stakeholders’ opinion in designing the initial questionnaire. Also, while reviewing websites of schools and colleges around the world, their experiences have been used in order to design the questionnaire.
- Calculating and reporting Kappa in order to check the stability between Delphi rounds and to decide on the completion of Delphi is one of the strong points of the methodology of this study.

## Conclusion

Different elements of a nursing education institution at the bachelor's level were determined. The results of this study can help related bodies to develop and implement a comprehensive and systematic evaluation. These results can also be a basis for making this model useful in other nursing courses or other fields.

### Supplementary Information

**Supplementary Material 1. **

**Supplementary Material 2. **

## Data Availability

The datasets used and/or analyzed during the current study are available from the corresponding author on reasonable request.
