# Supplementary material for: Adapting the Donabedian model in undergraduate nursing education: a modified Delphi study
Source: BMC Med Educ. 2024 Feb 27;24:202. doi: 10.1186/s12909-024-05187-7 (PMC10900582; doi:10.1186/s12909-024-05187-7)
Supplement: Supplementary file 1 — Supplementary Material 1. [file 12909_2024_5187_MOESM1_ESM.docx]

**Demographic information of participants and literature review in the major study.**

***Here we brought demographic information of those who participated in previous steps of the major study. This Delphi study is also a part of that major study. We used data from those previous steps to design the questionnaire in the first round of this Delphi study.***

**Four focus groups were held with nursing students:**

Four focus groups were conducted with the participation of bachelor’s, master’s, and Ph.D. nursing students. Of the 27 participants in these focus groups, 16 (59.3%) were female and 11 (40.7%) male. 77.8 percent of the participants (n=21) were studying for a Bachelor’s degree in nursing, 3.7 % (n=1) for a Master’s degree, and 18.5 % (n=5) for a Ph.D. The results of these focus groups are available in an article titled “What should be measured? Nursing education institutions performance: a qualitative study”([1](#_ENREF_1)).

**Seven individual interviews were held with faculty members and nurses who were involved in policymaking:**

The participants’ experiences included: Ministry of Health membership, faculty members, school/college management, hospital management, WHO regional experts network membership, national nursing research network membership, professional and scientific associations membership, insurance officer, accreditation committee membership, scientific journals’ editor in chief, scientific journal editorial team, clinical experiences, national nursing policy council membership, nursing board membership, school’s education development office membership. The mean years of work experience were 22 years. Five participants had Ph.D. degrees, and two participants had M.Sc. in nursing ([2](#_ENREF_2)).

**One focus group was held with clinical nurses:**

Six nurses participated in this focus group. They had an M.Sc. in nursing. They were working as a nurse in different wards in hospitals. And the mean years of experience were 11.16.

**One Google form was designed and filled out by 91 participants:**

91 participants filled out this form. 19.8% of whom were nursing students. 6.6% were nursing managers in hospitals. 31.9% were nursing faculty members. 1.1% were managers in nursing schools and 40.7% were nurses working in hospitals. The mean years of work experience were 12.7 years.

**A review was also conducted to identify the indicators available to measure the performance of nursing schools:**

We found 30 reports and web pages. Based on inclusion criteria, 20 were included in the data analysis ([2](#_ENREF_2)).

*Table S1: The titles of the documents that the reported indicators in them were used in the study and data analysis*

| **No** | **Title** |
| --- | --- |
| 1 | 2016 Performance Indicators (Lakehead University) ([3](#_ENREF_3)) |
| 2 | 2018-2019 Key Performance Indicators (Ontario Colleges) ([4](#_ENREF_4)) |
| 3 | The University of Arkansas at Monticello Academic Unit Annual Report, Academic Year: 2018-2019 ([5](#_ENREF_5)) |
| 4 | Academic Key Performance Indicators (KPI's) (Montana State University) ([6](#_ENREF_6)) |
| 5 | Nursing Program Specification (ALJOUF University College of Applied Medical Sciences) ([7](#_ENREF_7)) |
| 6 | College of nursing strategic plan 2015-2020 (University of Dammam) ([8](#_ENREF_8)) |
| 7 | Program Assessment (Chicago State University) ([9](#_ENREF_9)) |
| 8 | Ministry of Colleges and Universities Key Performance Indicators (University of Windsor) ([10](#_ENREF_10)) |
| 9 | Key performance Indicators Third Issue (2016-2013) (Sultan Qaboos University) ([11](#_ENREF_11)) |
| 10 | Key Performance Indicators (KPIs) 2017-2018 (Gadsden State Community College) ([12](#_ENREF_12)) |
| 11 | key performance indicators (University of the Virgin Islands) ([13](#_ENREF_13)) |
| 12 | Key Performance Indicators of the University of Hail (Definition-Patterns- Importance) ([14](#_ENREF_14)) |
| 13 | Key Performance Indicators (Georgian College) ([15](#_ENREF_15)) |
| 14 | Key Performance Indicators (Prairie State College) ([16](#_ENREF_16)) |
| 15 | Key Performance Indicators (Missouri State University) ([17](#_ENREF_17)) |
| 16 | Key Performance Indicators (Edge Hill University) ([18](#_ENREF_18)) |
| 17 | Key Performance Indicators (California Southern University) ([19](#_ENREF_19)) |
| 18 | Key Performance Indicator Dashboard (Mesa Community College) ([20](#_ENREF_20)) |
| 19 | Executive-Dashboard (Ball State University) ([21](#_ENREF_21)) |
| 20 | Curtin Annual Report 2020 (Curtin University) ([22](#_ENREF_22)) |

1. Ghofrani M, Valizadeh L, Zamanzadeh V, Ghahramanian A, Janati A, Taleghani F. What should be measured? Nursing education institutions performance: a qualitative study. BMJ Open. 2022;12(12):e063114.

2. Ghofrani M, Valizadeh L, Zamanzadeh V, Ghahramanian A, Janati A, Taleghani F. Baccalaureate nursing education institutions’ key performance indicators: a review of the existing indicators and qualitative analysis of expert interviews. BMC Nursing. 2023;22(1):357.

3. Lakehead University. 2016 Performance Indicators. Ontario, Canada: Lakehead University, 2016.

4. Ontario C. 2018-19 key performance indicators. Ontario, Canada: Colleges Ontario, 2019.

5. University of Arkansas. University of Arkansas at Monticello Academic Unit Annual Report. Arkansas, USA: School of Nursing (SON), 2019.

6. Office of Planning & Analysis. Academic Key Performance Indicators (KPIs) Montana, USA: Montana State University, 2017.

7. Education Evaluation Commission. Nursing Program Specification. Saudi Arabia: ALJOUF university College of Applied Medical Sciences, Department of nursing, Al-Qurayyat., 2018.

8. Hegazi M. College of nursing strategic plan 2015-2020. Dammam, Saudi Arabia: University of Dammam, 2016.

9. Chicago State University. Program Assessment. Chicago, USA: Department of nursing, 2021.

10. Office of Institutional Analysis. Ministry of Colleges and Universities Key Performance Indicators. Windsor, Canada: University of Windsor, 2020.

11. Planning & Statistics Department. Key performance Indicators Third Issue (2016-2013). Muscat, Oman: Sultan Qaboos University.

12. Gadsden State Community College. Key Performance Indicators (KPIs) 2017-2018. United States: Gadsden State Community College, 2018.

13. University of the Virgin Islands Planning Committee. key performance indicators. University of the Virgin Islands, 2020.

14. Quality and Development Department. Key Performance Indicators of the University of Hail (Definition-Patterns- Importance). Kingdom of Saudi Arabia.

15. Georgian College. Key Performance Indicators Ontario, Canada: Georgian College; 2021 [25/April/2021]. Available from: https://[www.georgiancollege.ca/about-georgian/corporate-information/key-performance-indicators/](http://www.georgiancollege.ca/about-georgian/corporate-information/key-performance-indicators/).

16. Operational Departments. Key Performance Indicators. Illinois, United States: Prairie State College, 2018.

17. Office of Institutional Research. Key Performance Indicators. Missouri, United States: Missouri State University, 2021.

18. Edge Hill University. Key Performance Indicators 2020 [updated 22/September/202013/April/2021]. Available from: https://[www.edgehill.ac.uk/corporate-information/board-of-governors/resources/section-b/key-performance-indicators/](http://www.edgehill.ac.uk/corporate-information/board-of-governors/resources/section-b/key-performance-indicators/).

19. California Southern University. Key Performance Indicators California, United States [28/April/2021]. Available from: https://[www.calsouthern.edu/key-performance-indicators/](http://www.calsouthern.edu/key-performance-indicators/).

20. MCC Office of Institutional Effectiveness. MCC Key Performance Indicators 2017-18 Arizona, United States: Mesa Community College.

21. Ball State University. executive-dashboard United States: Ball State University; 2019 [18/April/2022]. Available from: https://[www.bsu.edu/about/strategic-plan/executive-dashboard#accordion_question10](http://www.bsu.edu/about/strategic-plan/executive-dashboard#accordion_question10).

22. Annual Report 2020. Australia: Curtin University, 2021.
