# Supplementary material for: Adapting the Donabedian model in undergraduate nursing education: a modified Delphi study
Source: BMC Med Educ. 2024 Feb 27;24:202. doi: 10.1186/s12909-024-05187-7 (PMC10900582; doi:10.1186/s12909-024-05187-7)
Supplement: Supplementary file 2 — Supplementary Material 2. [file 12909_2024_5187_MOESM2_ESM.docx]

**Table S2:** Components, key elements, and related items for adapted Donabedian Model in nursing education

During a 3-round Modified Delphi study related elements and items are confirmed by an expert panel consisting of 13 nursing experts.

| **Component** | **Key Elements** | **Items** | No. |
| --- | --- | --- | --- |
| Structure | Staff (academic and non-academic) | Training courses and workshops held for teachers and other staff | 1 |
|  |  | Human resources standards | 2 |
|  |  | Reward systems, payment methods | 3 |
|  | Equipment | Non-digital/digital library (books, theses/dissertations, journals) | 4 |
|  |  | Laboratories and laboratory equipment | 5 |
|  |  | Online teaching equipment | 6 |
|  |  | Information Technology unit | 7 |
|  |  | Education aids equipment and moulages | 8 |
|  |  | Physical equipment of classes | 9 |
|  | Guidelines | Curriculum | 10 |
|  |  | Lesson plans | 11 |
|  | Resources and facilities | Research centers affiliated with the school/university | 12 |
|  |  | Clinical training field affiliated with the university/school | 13 |
|  |  | Recreation and health facilities available for students, teachers, and other staff | 14 |
|  |  | School website | 15 |
|  |  | Financial resources | 16 |
|  |  | Standards of educational and study environments | 17 |
|  | Students demographics and characteristics | Diversity of students (domestic and international) | 18 |
|  |  | Assessing of applying students (personal and academic characteristics) | 19 |
| Process | Communication | Holding meetings between different departments of the school | 20 |
|  |  | Holding interdisciplinary meetings | 21 |
|  |  | Holding intersectional meetings (community, hospitals,...) | 22 |
|  |  | International communication | 23 |
|  | Education | Courses and workshops for students | 24 |
|  |  | Continuing education courses delivered | 25 |
|  |  | Teaching theoretical and practical courses (teaching skills and methods, scientific-clinical-moral qualification of teachers-number of teaching and training hours provided) | 26 |
|  |  | Familiarizing students with the rules and processes of the institution | 27 |
|  | Evaluation | Evaluation of teaching staff | 28 |
|  |  | Evaluation of students | 29 |
|  |  | Evaluation of school departments | 30 |
|  |  | Evaluation of non-teaching staff | 31 |
|  |  | Internal accreditation | 32 |
|  | Cooperation | Holding national and international conferences and congress | 33 |
|  |  | Implementation of joint educational/research/service/production projects with other schools and industry | 34 |
|  |  | Participation in projects related to the promotion of community health | 35 |
|  | Consultation | Counseling with students (personal-educational) | 36 |
| Outcome | Knowledge development | Books and articles published by the school staff and the number of citations to them | 37 |
|  |  | Papers presented at national and international conferences | 38 |
|  |  | Innovations (developing of nursing models, etc.) | 39 |
|  | Nursing image | Nursing image | 40 |
|  | Alumni’s outcomes | Clinical competence | 41 |
|  |  | Professional knowledge | 42 |
|  |  | Higher education | 43 |
|  |  | Employment in nursing | 44 |
|  |  | Professional and organizational commitment | 45 |
|  |  | Interest in the nursing profession | 46 |
|  | Students outcomes | Theoretical and practical learning | 47 |
|  |  | Scientific-cultural-artistic-sports achievements | 48 |
|  |  | Professional and organizational commitment | 49 |
|  |  | Interest in the nursing profession | 50 |
|  |  | Student's physical and mental health | 51 |
|  |  | Students transfer | 52 |
|  |  | Dropout | 53 |
|  | Related clinical setting’s performance | Nursing care quality | 54 |
|  | Accreditation and evaluation results | National and international ranking of the school | 55 |
|  |  | Awards won (school-staff) | 56 |
|  | Satisfaction | Teachers | 57 |
|  |  | Students | 58 |
|  |  | Hospital personnel and managers | 59 |
|  |  | Recipients of nursing services | 60 |
